# Supplementary material for: The mediating role of positive orientation in the relationship between personality traits and attitudes towards artificial intelligence
Source: PLoS One. 2026 Mar 11;21(3):e0342317. doi: 10.1371/journal.pone.0342317 (PMC12978485; doi:10.1371/journal.pone.0342317)
Supplement: Attachment — Submitted filename: Response to Reviewers.doc [file pone.0342317.s002.doc]

**Response to Reviewers**

| **Note from the reviewer** | **Response** | **Change in the article** |
| --- | --- | --- |
| Please ensure that your manuscript meets PLOS ONE's style requirements, including those for file naming. | Done |  |
| Please state what role the funders took in the study. | Added sentence: | The funder had no role in study design, data collection and analysis, decision to publish, or preparation of the manuscript. |
| At this time, please upload the minimal data set necessary to replicate your study's findings to a stable, public repository (such as figshare or Dryad) and provide us with the relevant URLs, DOIs, or accession numbers that may be used to access these data. | Data are at public repository: 10.6084/m9.figshare.29873630  Added re-new sentences and link: | The data used for this article can be accessed at 10.6084/m9.figshare.29873630      All the data have been made publicly available at the Figshare and can be accessed at 10.6084/m9.figshare.29873630. |
| 1. Reconstruction and deepening of the discussion The discussion needs a better structure – key findings (especially mediation hypotheses) should be clearly highlighted at the beginning of this section. The following should be explained in detail and conceptually: the lack of mediation for openness – with possible theoretical interpretations, the suppression effect for conciliatoriness – which is unclear in its current form. Please also consider the potential risks associated with an overly positive attitude towards AI, as mentioned by both reviewers. | Done |  |
| 2. Transparency of methods and data Descriptions of tools must be complete: full name, authors, application, interpretation of results. |  | Description of the methods has been changed |
| Table 1 must include: mean values and standard deviations, demographic variables (age, gender, place of residence, education), full correlation matrix (“all-against-all”). |  | Table 1 has been changed |
| 3. Limitations of the study The limitations associated with the use of TIPI, especially for openness, should be clearly indicated. Please add information that the actual use of AI has not been investigated and treat this as a clear limitation.  Please also note the limited possibility of generalizing the results beyond the Polish context. | Added:  Already noted:  The conclusions drawn in the present study are contingent upon the characteristics of the sample and the measurement methods used. By focusing on a singular population from Poland, it may not be possible to recognise the constraints involved in generalising findings to broader, global contexts. The unique sociocultural landscape may shape individuals' attitudes towards AI differently than in other regions. Individuals from diverse educational backgrounds may exhibit differing levels of technological apprehension, which could influence their attitudes towards AI [8]. | The use of the Ten Item Personality Inventory (TIPI) entails certain psychometric constraints, particularly with regard to the measurement of openness to experience. Although TIPI offers the advantage of brevity, its reduced number of items may limit the reliability and depth of construct assessment compared to longer, more comprehensive personality inventories. It is important to note that the actual use of artificial intelligence (AI) by the participants was not examined. The study focused on self-reported attitudes and characteristics rather than on direct behavioral measures of AI adoption or interaction. This absence of behavioral data constitutes a clear limitation, as it restricts the ability to draw conclusions about real-world AI use. |
| 4. Language and style The text requires linguistic and editorial revision. There are some complex, unclear sentences and redundancies. I recommend seeking the assistance of a professional language editor before resubmitting the revised version. | Professional proofreading of the text was done |  |
|  |  |  |
| Please include the full names of the measurement scales in the abstract. |  | Full names of the methods were added in the abstract |
| Additionally, please state in the abstract that the hypotheses were only partially confirmed — specifically, the hypothesis regarding openness was not supported. | The sentence in the abstract has been changed. | The mediating effect of positive orientation on the relationship between personality traits and attitudes towards artificial intelligence was partially confirmed. |
| The sentence "Park and Woo [6] reported that extraversion was associated with negative emotions and low functionality, whereas agreeableness was linked to both positive and negative emotions, as well as high sociality and functionality.” needs to be rephrased, as in its current form it suggests a positive correlation between extraversion and negative emotions. The subsequent sentences should also be clarified to indicate that they refer to attitudes towards AI. | Corrected | Park and Woo [11] reported that extraversion was associated with negative emotions about AI and low functionality of AI. **Agreeableness** correlated with **both positive and negative emotions** toward AI and was linked to higher perceived **sociality** and **functionality** of AI. **Conscientiousness** was associated with **fewer negative emotions** and higher **perceived functionality,** but **lower perceived sociality.**The results indicated that neuroticism was associated with negative emotions about AI and high sociality of AI, whereas openness was positively linked to functionality of AI. A consistent finding was that personal innovativeness in information technology was associated with positive attitudes towards AI. |
| It would be beneficial to include a brief review of studies on the relationship between positive orientation and the Big Five personality traits in the Introduction. | Added: | Research on healthy adult populations has consistently demonstrated that this disposition is systematically related to the Big Five personality traits. In multiple cross-sectional studies, extraversion and emotional stability (low neuroticism) emerge as the strongest positive correlates of positive orientation [18, 19]. High extraversion, characterized by sociability, positive affect, and energetic engagement with the environment, supports an optimistic and satisfied view of life. Similarly, emotional stability reduces susceptibility to negative emotions, thereby reinforcing a generally positive outlook. Conscientiousness is also positively associated with positive orientation [19]. Individuals high in conscientiousness tend to set goals, plan effectively, and persevere in the face of challenges—behaviors that contribute to life satisfaction and optimism. Agreeableness correlates with positive orientation through prosocial attitudes, empathy, and cooperative behaviors, which foster supportive social relationships and enhance well-being [19]. The association between openness to experience and positive orientation is more variable, but studies in adult samples have reported positive links [18, 19]. This relationship may stem from cognitive flexibility, curiosity, and appreciation for novelty, traits that can help individuals find meaning and satisfaction in diverse life experiences. Overall, findings from healthy adult samples across different cultural contexts indicate that positive orientation is not an isolated construct but is embedded within the broader personality system described by the Big Five. These associations highlight the role of enduring personality traits in shaping a consistently positive perspective on life and oneself [18, 19]. |
| Furthermore, the introduction should begin with several arguments highlighting the potential negative consequences of adverse attitudes towards AI. It would also be valuable to mention examples of negative consequences resulting from overly positive and uncritical attitudes towards AI. | Added: | Negative attitudes toward artificial intelligence (AI) can have far-reaching consequences for technology adoption and societal progress. Research shows that distrust or skepticism toward AI may reduce individuals’ willingness to use AI-based tools, hinder the implementation of innovative systems, and limit potential benefits in areas such as healthcare, education, and public administration [2, 3]. Such resistance can slow organizational efficiency and exacerbate existing inequalities by widening the digital divide between those who adopt AI and those who reject it. Moreover, fear of job displacement and perceived threats to privacy or autonomy may intensify negative affect toward AI, further reducing engagement with its applications [4].  However, excessively positive and uncritical attitudes toward AI also carry significant risks. Studies on automation bias demonstrate that overreliance on algorithmic recommendations can impair human judgment, leading to the acceptance of incorrect outputs without sufficient verification [5]. Uncritical acceptance of AI outputs may also facilitate the perpetuation of embedded biases, resulting in unfair or discriminatory outcomes in domains like recruitment or criminal justice [6]. These findings underscore the need for balanced, informed attitudes toward AI that combine openness to innovation with critical evaluation of potential limitations and harms. |
| In the Methods section, please start each instrument description with its full name, the authors, and the purpose for which the tool was used in this study. Clearly explain what high scores on each scale indicate. | Done |  |
| In Table 1, please add information about the means and standard deviations for each variable.  Also, include sociodemographic variables such as age, gender, place of residence, and education.  Table 1 should be structured to allow for “all-against-all” correlations — that is, all variables should appear both in columns and rows. | Done |  |
| Were participants asked whether they use AI, how frequently, and for what purpose? If not, please mention this as a limitation of the current study. | Done |  |
| The sentence “In the aforementioned model, a substantial indirect effect was obtained (B = .243, SE = .063, 95CI (0.123, 0.369)), indicating that positive orientation serves as a significant mediator” — please remove the word “significant”, as there is no such thing as a “non-significant mediator.” This correction should be applied throughout the descriptions of analyses. | Done |  |
| The Discussion section should begin with the most important results, i.e., those related to the mediation hypothesis.  Please elaborate further on the lack of mediation for openness. Provide additional interpretations — for example, that openness is the personality trait least associated with positive orientation, likely because openness to experience involves openness to both positive and negative experiences. Individuals high in openness may exhibit ambivalent attitudes toward AI — strongly positive in some respects, but also negative, as they may perceive the potential threats associated with AI’s power.  Please also address in the discussion the potential risks of overly positive attitudes toward AI.  Add a section discussing practical implications of the study's findings in psychological practice.  Finally, include a conclusion at the end of the discussion | Added: | The present study examined the mediating role of positive orientation in the relationship between personality traits and attitudes toward artificial intelligence (AI). The findings partially confirmed the proposed model: positive orientation significantly mediated the effects of extraversion, agreeableness, conscientiousness, and neuroticism on attitudes toward AI, while no mediation was observed for openness to experience. Specifically, higher levels of extraversion and conscientiousness were associated with greater positive orientation, which in turn predicted more favourable attitudes toward AI. Conversely, neuroticism showed a negative indirect effect, indicating that positive orientation buffered the detrimental impact of emotional instability on AI acceptance. Interestingly, the analysis revealed a suppression effect for agreeableness, suggesting a more complex pattern in which positive orientation conceals an underlying negative association between agreeableness and attitudes toward AI.  As noted by Stein et al. [9], openness is associated with intellectual curiosity and critical evaluation of new technologies rather than with uniformly positive attitudes. Individuals high in openness may therefore approach AI with greater reflection and ambivalence, balancing interest in innovation with awareness of its potential risks.  While promoting positive orientation can facilitate openness toward AI, it is equally important to acknowledge the potential risks of overly positive attitudes. Excessive optimism or uncritical acceptance of AI systems may lead individuals to underestimate ethical, privacy, or safety concerns, and to overtrust algorithmic decisions without adequate scrutiny. As noted in prior studies, automation bias and techno-enthusiasm can reduce users’ willingness to question AI outputs, thereby increasing the likelihood of errors or unfair outcomes in applied settings [5, 6]. Consequently, efforts to enhance AI acceptance should aim to balance optimism with critical awareness, fostering what might be termed ‘informed trust’—a stance that combines openness to innovation with careful evaluation of its limitations and societal implications.  In conclusion, the present study demonstrates that personality traits, particularly when filtered through the lens of positive orientation, play a meaningful role in shaping attitudes toward artificial intelligence. Positive orientation emerges as a key psychological mechanism that fosters openness to technological change while buffering negative emotional tendencies such as anxiety or distrust. At the same time, the findings highlight the importance of maintaining a balanced perspective—encouraging optimism toward AI while avoiding uncritical acceptance. By integrating individual~~,~~ emotional factors, this study contributes to a more comprehensive understanding of the psychological foundations of AI acceptance and offers practical insights for education, communication, and policy aimed at promoting responsible and informed engagement with emerging technologies. |
|  |  |  |
| Clarify the Role of Suppression in Agreeableness: The finding of a suppression effect involving agreeableness is both statistically and theoretically interesting. However, the explanation of suppression could be expanded with greater conceptual clarity. Currently, the discussion is dense and somewhat circular. A clearer exposition on how suppression differs from full or partial mediation—and its implications for interpreting the negative direct effect—would improve accessibility for a broader readership. | Added: | This effect indicates a more complex statistical relationship than simple mediation. In classical mediation, a mediator transmits part or all of the effect of an independent variable on a dependent variable, resulting in a reduction of the direct relationship once the mediator is included. In contrast, suppression occurs when the inclusion of a third variable—here, positive orientation—reveals or strengthens a hidden relationship that was previously obscured. In the present study, the direct relationship between agreeableness and attitudes towards AI was initially nonsignificant but became significant and negative after accounting for positive orientation. This suggests that positive orientation shares variance with agreeableness that conceals their opposing associations with attitudes toward AI.  Conceptually, this finding implies that although agreeableness is generally linked to prosociality, cooperation, and trust, these characteristics may also predispose individuals to be more cautious or morally concerned about emerging technologies. Positive orientation, encompassing optimism and life satisfaction, may counterbalance this cautious tendency, masking the underlying scepticism associated with higher agreeableness. Once positive orientation is statistically controlled, the more critical or apprehensive aspect of agreeableness becomes visible. This pattern underscores that suppression effects do not simply indicate statistical artefacts but can illuminate opposing psychological mechanisms within personality traits, offering a more nuanced understanding of how prosocial dispositions interact with emotional outlooks in shaping AI acceptance. |
| Address TIPI Limitations: While the use of the Ten Item Personality Inventory (TIPI) is defensible due to space and time constraints in survey research, the limitations of such brief measures (e.g., low internal consistency, particularly for traits like openness) should be acknowledged more explicitly in the limitations section. This is particularly pertinent given the non-significant effects associated with openness to experience. | Done |  |
| Broaden Discussion of Cross-Cultural Generalizability: The discussion section rightly acknowledges the study's national scope (Poland), but the broader implications for cross-cultural variability in AI attitudes could be better elaborated. The sociocultural specificity of AI narratives may influence the mediational pathways observed and should be reflected more critically. | Added: | The generalizability of the present findings beyond the Polish context warrants careful consideration. Attitudes toward AI are shaped not only by individual dispositions but also by culturally embedded narratives concerning technology, progress, and human agency. Recent research indicates that cross-cultural variability in AI perceptions is substantial, with trust, moral acceptability, and perceived usefulness differing systematically across societies [37, 38, 39]. Cultural frameworks—such as individualism versus collectivism, secularism versus religiosity, or levels of institutional trust—can moderate the relationship between psychological traits and attitudes toward technological innovation [37, 41]. In cultures characterised by higher social and institutional trust, positive orientation may act as a catalyst that amplifies openness to AI, whereas in societies marked by ambivalence toward authority or concerns about technological displacement, the same disposition might serve more as an adaptive buffer than a driver of acceptance [38, 40]. From this perspective, the Polish results may reflect a sociocultural context of **cautious optimism**, where technological advancement is recognised as valuable yet approached with ethical reflection and concern for social consequences. Polish surveys indicate that while the public generally supports digitalisation and recognises the benefits of AI, there remains notable scepticism regarding automation and potential threats to employment and privacy [42, 43]. Such a climate may have shaped the mediational pattern observed—where positive orientation compensates for underlying uncertainty rather than merely amplifying enthusiasm. Consequently, the current findings may illustrate how culturally specific narratives about AI interact with personality-based dispositions to form nuanced, context-dependent attitudes. Recognising these dynamics opens a path for comparative research that explores whether the observed mediation by positive orientation represents a universal psychological process or a culturally contingent adaptation. |
| Language and Style Minor Edits: While the manuscript is generally well-written, a few grammatical and syntactic issues remain. For example, there are occasional redundancies and overly long sentences in the discussion that could be edited for clarity. A professional language edit may enhance readability, particularly for an international audience. | Professional proofreading of the text was done |  |
| Expand on Practical Implications: The conclusion alludes to the potential for using positive orientation as a lever for improving AI acceptance. It would be helpful to suggest concrete pathways for doing so, e.g., through education, optimism-based interventions, or communication strategies, drawing on existing psychological literature. | Added: | Promoting positive orientation offers a practical avenue for enhancing the acceptance of artificial intelligence in society. Drawing on existing psychological literature on positive orientation and well-being [18, 34, 35], educational initiatives that incorporate elements of positive psychology—such as optimism training, self-efficacy development, and resilience-building—may foster greater openness to technological innovation. Programs aimed at increasing AI literacy could highlight both the potential benefits of AI and the mechanisms that ensure its ethical and safe use, thereby reducing uncertainty and fear. Encouraging individuals to adopt a constructive, future-oriented mindset may strengthen their ability to adapt to technological changes and perceive AI as a source of opportunity rather than a threat.  Furthermore, communication strategies that frame AI as a supportive tool designed to augment rather than replace human capabilities may align with individuals’ positive orientation and promote trust. Transparent and balanced messaging—emphasizing both the opportunities and limitations of AI—can reinforce realistic optimism, a concept widely discussed in the literature on adaptive functioning and well-being [36, 34]. Drawing on insights from positive psychology and communication research, policymakers, educators, and technology developers can thus design interventions that simultaneously enhance optimism, awareness, and critical reflection—factors essential for fostering balanced and informed acceptance of AI. |
